# Multispectral sensor fusion in SmartWatch for in situ continuous monitoring of human skin hydration and body sweat loss

**DOI:** 10.1038/s41598-023-40339-7

**Published:** 2023-08-17

**Authors:** Elena Volkova, Alexey Perchik, Konstantin Pavlov, Evgenii Nikolaev, Alexey Ayuev, Jaehyuck Park, Namseok Chang, Wonseok Lee, Justin Younghyun Kim, Alexander Doronin, Maksim Vilenskii

**Affiliations:** 1 Sensor Solutions Laboratory, Samsung R&D Institute Russia, 127018 Moscow, Russia; 2 Health H/W R&D Group, Samsung Electronics, Suwon, 16678 Korea; 3 School of Engineering and Computer Science, Victoria University of Wellington, 6140 Wellington, New Zealand

**Keywords:** Biophotonics, Quality of life

## Abstract

Post-pandemic health operations have become a near-term reality, discussions around wearables are on the rise. How do wearable health solutions effectively deploy and use this opportunity to fill the gap between wellness and healthcare? In this paper, we will talk about wearable healthcare diagnosis, with a particular focus on monitoring skin hydration using optical multi-wavelength sensor fusion. Continuous monitoring of human skin hydration is a task of paramount importance for maintaining water loss dynamics for fitness lovers as well as for skin beauty, integrity and the health of the entire body. Preserving the appropriate levels of hydration ensures consistency of weight, positively affects psychological state, and proven to result in a decrease in blood pressure as well as the levels of “bad” cholesterol while slowing down the aging processes. Traditional methods for determining the state of water content in the skin do not allow continuous and non-invasive monitoring, which is required for variety of consumer, clinical and cosmetic applications. We present novel sensing technology and a pipeline for capturing, modeling and analysis of the skin hydration phenomena and associated changes therein. By expanding sensing capabilities built into the SmartWatch sensor and combining them with advanced modeling and Machine Learning (ML) algorithms, we identified several important characteristics of photoplethysmography (PPG) signal and spectral sensitivity corresponding to dynamics of skin water content. In a hardware aspect, we newly propose the expansion of SmartWatch capabilities with InfraRed light sources equipped with wavelengths of 970 nm and 1450 nm. Evaluation of the accuracy and characteristics of PPG sensors has been performed with biomedical optics-based simulation framework using Monte Carlo simulations. We performed rigorous validation of the developed technology using experimental and clinical studies. The developed pipeline serves as a tool in the ongoing studies of the next generation of optical sensing technology.

## Introduction

Since 1977, when the world's first wireless heartrate (HR) monitor consisting of a chest strap transmitter with a wrist-worn receiver was introduced by PolarElectroto^1^ gave the athletes “real-time feedback during the exercise” a number of notable achievements in wearable technology has been made. During the past decade, Samsung introduced an advanced Smart Bio-Processor, a system on a chip (SoC), that measures body fat, and skeletal muscle mass, heart rate (HR), heart rhythm, skin temperature and stress level for wearable devices^2^ Nowadays, our Smart watches are equipped with Samsung’s BioActive Sensor^3^ which has evolved to become far more advanced and miniature technology and not only able to “just” count HR, steps and calories, but also can monitor sleep, measure blood pressure and more^4–8^ The backbone and the main driver of this notable success is the PPGtechnology which is now widely used in the continuous monitoring of health status^9^ due to the convenient location of the sensor in a smartwatch/fitness bracelet on the wrist^10^. Utilization of PPG for health and fitness monitoring have attracted considerable consumer interest over the past few years. At present, wearable devices, including smart watches and fitness trackers, routinely monitor/analyze the PPG signal and provide non-invasive information of the aforementioned human health indicators with recent additions of temperature, blood oxygen level (SpO_2_), etc. monitoring capabilities^11,12^. PPG signal is used to assess stress and sleep patterns^13^. Currently, atrial fibrillation (A-fib) can be routinely detected using PPG from a smart watch^14^. It has been demonstrated how a smart phone application can assist with determination of vascular aging associated with increased arterial stiffness^15^. On the other hand, notable advances have been made with SmartWatch-assisted planning and exercising of a particular fitness program which often requires the knowledge of the basic physiological parameters of a consumer. For instance, the information about pulse zones makes it possible to evaluate the efficiency of oxygen delivery through the circulatory system to exercising muscles. Long-term monitoring of user's workouts allows the SmartWatch to create effective personal training plan. Continuous tracking of a user's exercise routine empowers SmartWatch applications to create efficient, personalized fitness plans. For such case, it is important to continuously monitor the PPG signal and quickly receive notifications when the specified boundaries of the pulse zone are breached.

**Figure 1:**
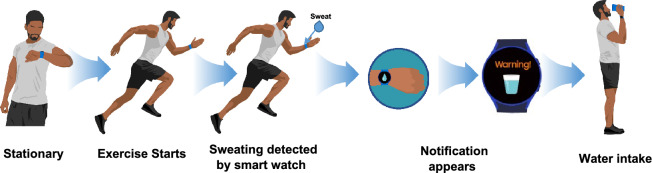
Schematic presentation of the concept of SmartWatch technology advising consumer of the need to rehydrate e.g. due to water loss after training/exercise.

Nevertheless, in this work we would like to take a step forward into perplexing and often unpredictable terra incognita of the real time continuous monitoring of body water loss. We would like to demonstrate that by adding certain spectral selectivity functionality to PPG this task becomes feasible. In the previous studies, utilization of PPG for continuous monitoring of human body hydration dynamic has been largely avoided. Once successfully implemented in the wearables, it has a high potential of becoming a topical health, beauty and exercise application (Fig. 1). Indeed, regular water-intake regime is incredibly important for the correct functioning of organs and maintain the internal balance of the human body. Each chemical reaction, including the production of energy or the process of decomposition and glucose storage, requires a certain amount of water. Dehydration negatively affects exercise performance, thermoregulation, and cardiovascular response^16,17^.

During intense physical activity, human body loses most of the fluid through sweating. At the same time, thermal energy that muscle cells produce is spent on heating the blood. Water from the blood fills the sweat glands under the influence of the temperature increase^17^. The process of evaporation of the liquid leads not only to the cooling of the body, but also to a decrease in blood volume. In this connection, the amount of fluid in the body must be replenished in time, otherwise the process of thermoregulation is disturbed and heat exhaustion may occur. For example: heat exhaustion is the excessive loss of salts (electrolytes) and fluids, it difficult for the body to maintain a healthy core temperature. The heat that occurs during physical activity is not compensated by sweating, but accumulates in the body, which leads to an increase in internal temperature and a deterioration in physical condition.

Generally, thirst is a precise mechanism for regulating water and electrolytes in the body. Thirst occurs when osmolality and the concentration of sodium ions in blood plasma increase as a result of sweating^18^. These changes are perceived by hypothalamus receptors responsible for maintaining homeostasis, and in particular for maintaining the osmotic pressure of blood plasma. Hypothalamic hormones stimulate an increase in water and sodium reabsorption, and reflex stimulation of the cingulate gyrus leads to the formation of a feeling of thirst, forcing a person to drink^18^. An increase in thirst reduces the effectiveness of exercise, thereby reducing the possible loss of fluid through sweating. However, during the state of an active exercise, thirst becomes an insufficient indicator of the status of a lack of water in the body. Thirst is usually not perceived by a person, with loss of body weight to 1–2% due to water, while working capacity and endurance are already beginning to diminish significantly^17^. The lack of water in the body causes a feeling of dry mouth, weakness, fatigue, irritability and headache. Monitoring of body hydration and timely fluid intake is necessary to achieve high results and good health. During physical activity, an athlete may experience “voluntary dehydration” or a delayed reaction of the body to a lack of water. Drinking balance involves choosing the optimal amount of fluid that should be consumed during training. Excess fluid in the body is also undesirable because it can lead to hyponatremia. The body considers that there is too much water in the blood and sends some of it to other organs. An increase in fluid volume by 2% can lead to generalized edema, decreased physical activity and disruption of the human brain. Therefore, monitoring the body's hydration and timely fluid intake during sports is crucial to achieve high results and maintain good physical shape of the body.

## Results

**Figure 2:**
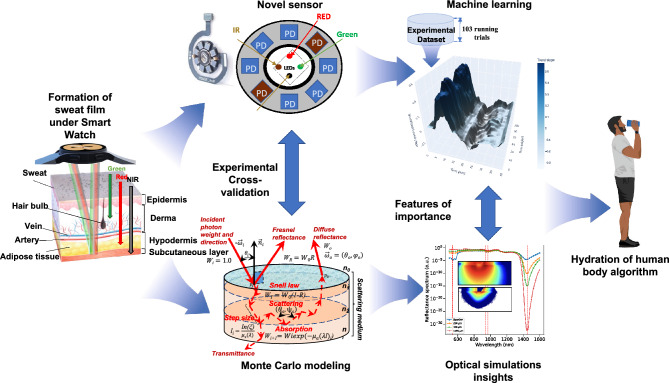
Our capture-to-sensing pipeline. Here: a sweat film is formed under the SmartWatch prototype; Custom-made sensor evaluates change in PPG signal at a range of wavelengths and distances; The trends in the signal are investigated using experimentally validated numerical algorithms of light transport in skin; Particular features of importance due to fluctuations of wet/dry skin optical properties are extracted based on their significance and a ML method is trained to readily detect them; Finally, sweat loss is quantitatively accessed and recommendations to consumer are made.

Within a framework of this research, we created a complete capturing, modeling and analysis pipeline (depicted in Fig. 2) specifically tailored to access hydration proprieties of skin with SmartWatch in order to identify the exact moment when sweating occurs. Our models are aided by direct measurements of tissue's functional properties using custom-tailored sensor. By combining powerful Machine Learning (ML) techniques, state-of-the-art numerical simulation algorithms for photon transport in biological techniques and clinical studies we achieved near-instantaneous evaluations of human skin PPG signals and their changes related to different stages of sweating. We performed rigorous validation of the developed pipeline by comparing with measurements obtained using laboratory grade hardware in vivo. The developed framework serves as a tool in the ongoing internal development of the next generation of sensing capabilities.

### Novel hardware design and in silico experimentation

**Figure 3:**
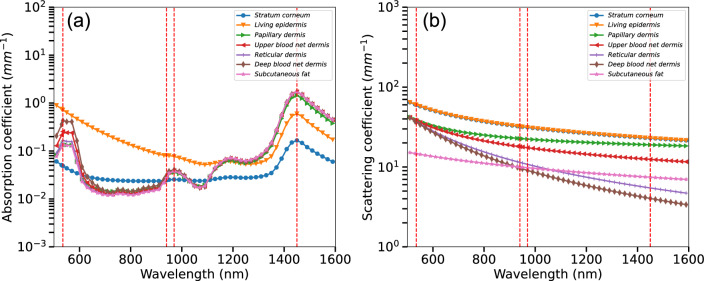
Optical properties of human skin and proposed sensor configuration. Here, (**a**)—absorption coefficients of key skin tissues chromophores including melanin, oxy-hemoglobin, deoxy-hemoglobin, baseline and water; (**b**)—scattering coefficients of the functional tissue layers.

When applied to human skin, PPG signal formation is mandated by light absorption and scattering within the tissue due to blood, melanin, collagen, water and other pigments as well as the specular reflection at skin-air interface^19,20^ (Fig. 3a, b). For instance, notable bands and corresponding absorptions/scattering effects for melanin, blood hemoglobin at 416, 542, and 575 nm, and water at 980 nm have been extensively studied in the past^18–20^. In other words, spectral composition of the light penetrating through biological tissues largely depends on the concentration and spatial distribution of chromophores within the skin. When applied to a practical wearable application, the accuracy of the registration of the PPG signal largely depends on multiple other factors which include but not limited to: sensor geometry and its position, band stiffness and environmental factors such as temperature, humidity, etc. Moreover, the optimal parameters of the sensor such as its geometry, numerical aperture, source-detector shape and separation, wavelengths, etc. require careful selection and the most optimal settings need to be accurately studied and their performance evaluated. This step is feasibly performed in silico by means of numerical simulations and involves computing the light-tissue interactions which are formally described by the Radiative Transfer Equation (RTE). The RTE theory originates from the point of energy conservation and serves as a basis for photometry^20–22^. This theory has been extensively used in a number of studies including atmospheric and ocean scattering, astrophysics, and subsequently biomedical optics. Numerous attempts were made in the past to evaluate physiological properties of the tissue and connect them with the diffusive scatted and absorbed optical radiation^23–30^. The macroscopic energy balance and statistical average of light transport and their energy conservation through the scattering and absorptive media at equilibrium is described as:

1 $$\left( {\vec{\omega } \cdot \vec{\nabla }} \right)L\left( {{\varvec{p}},\vec{\omega }} \right) = - \left( {\mu_{s} \left( \lambda \right) + \mu_{a} \left( \lambda \right)} \right)L\left( {{\varvec{p}},\vec{\omega }} \right) + \mu_{s} \left( \lambda \right)\mathop{{\int\!\!\!\!\!\int}\mkern-21mu \bigcirc} {p\left( {\vec{\omega },\vec{\omega }^{\prime}} \right)L\left( {{\varvec{p}},\vec{\omega }^{\prime}} \right)d\omega^{\prime} + Q\left( {{\varvec{p}},\vec{\omega }} \right)}$$

Here, $L\left( {\varvec{p}},\overrightarrow{\omega }\right)$ refers to the energy radiance in the medium at a specific point ${\varvec{p}}$ in the direction $\overrightarrow{\omega }$, where ${\mu }_{s}\left(\lambda \right)$ and ${\mu }_{a}\left(\lambda \right)$ are the spectrally-resolved scattering and absorption coefficients, $p\left(\overrightarrow{\omega } ,\overrightarrow{\omega }{\prime}\right)$ corresponds to the scattering phase function and $Q({\varvec{p}},\overrightarrow{\omega })$ represents the optical radiation source function, respectively. $\overrightarrow{\nabla } L$ represents the spatial gradient of the radiance telling how much radiance changes per unit distance. For homogeneous, single-layered materials, the RTE is usually solved by analytical methods such as diffusion approximation. Nevertheless, due to the complex and inhomogeneous structure of human skin, no general analytical solution to RTE for our sensor configuration exists that can describe the detected signal and how it is affected by its structural or physiological changes.

Fortunately, there is an example of a stochastic alternatives: the Monte Carlo (MC) method which has been, throughout the years, a tool-of-choice for the assessment of optical radiation propagation and spatial localization of signals in biological tissues in the field of biomedical optical diagnostics. MC method was first introduced for simulation of light propagation in biological tissue in 1983 by Wilson and Adam^31^. Subsequently, the MC has been further developed by multiple research groups and is now utilized widely in Biomedical Optics^31–35^. The MC is now considered a “gold standard” and a convenient tool for modeling signals due to the possibility of accounting for the complex structure of the object under study, the boundary conditions, the geometry of the probe beam and other features. MC enables a direct comparison between simulated and experimental results, as well as predicting the outcomes of future measurements when a sufficiently large number of statistical data is accumulated. However, the accuracy of such modeling is determined by the cost of machine time (i.e. the method is highly resource consuming), as well as the correspondence of the model to the simulated object. Therefore, a direct application of MC method was not adopted widely in wearables due to MC’s notorious computational inefficiency, the lack of domain-specific knowledge in tissue biology/optics and a number of constrains related to dynamic nature of PPG signal acquisition.

**Figure 4:**
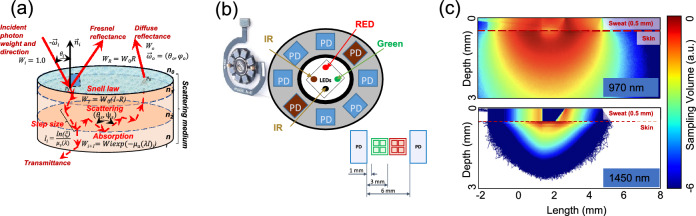
MC simulation procedure and geometrical configuration of the developed sensor. Schematic presentation of MC simulation procedure, (**a**) geometrical configuration of the developed sensor, (**b**) example MC simulations of optical signal propagation for the extended SmartWatch sensor configuration in human skin with topical water/sweat layer at 970 nm (top) and 1450 nm (bottom), (**c**).

MC method is based on modeling energy transfer through the medium and the corresponding principles have been described comprehensively elsewhere^22^. Briefly, multiple so-called photon packets are first assigned with statistical unit weight ${W}_{0}$ and injected into a modeling medium (Fig. 4a). The packets undergo a sequence of randomly-sampled events representing light-media interactions (e.g. scattering, absorption, reflection, refraction and media layer transfer at the boundaries). The path length distribution $p\left(l\right)$ for a photon packet propagating distance $l$ between scattering events is determined randomly and follows the Beer–Lambert law as $p\left(l\right)={\upmu }_{t}\left(\uplambda \right){e}^{-{\upmu }_{t}\left(\uplambda \right)l}$. Subsequently, the photon packet position is updated as ${{\varvec{p}}}_{\text{i}}={{\varvec{p}}}_{{\text{i}}-{1}}+\overrightarrow{{\upomega }_{i}{\prime}}{l}_{\text{i}}$ and its statistical weight is scaled by absorption ${W}_{i}={W}_{i-1}{e}^{-{\upmu }_{a}\left(\uplambda \right)l}.$ A new direction of the photon packet $\overrightarrow{{\upomega }_{i}{\prime}}$ is determined at each scattering event using a phase function of choice e.g. the Henyey–Greenstein function:

2 $$p_{HG} \left( {cos\left( {\uptheta } \right)} \right) = \frac{1}{{4{\uppi }}}\frac{{1 - g^{2} }}{{\left( {1 + g^{2} - 2gcos\left( {\uptheta } \right)} \right)^{3/2} }},$$

where $g$ is the anisotropy factor. The input parameters when applying this method are the optical properties and geometry of the medium, which determine the lengths and forms of individual photon trajectories.

Based on the extensive knowledge of light-tissue interaction and in silico experimentation, we have designed and produced the actual hardware prototype of a novel multi-wavelength optical sensor which has been currently built into the common Samsung Galaxy Watch Active 2 device therefore extending its capabilities extensively. One of the main futures of the sensor is its prolonged spectral sensing range. Complimentary to the two existing 535 and 645 nm sensors two wavelengths (970 and 1450 nm), have been added in order to enable signal acquisition in the Near-infrared (NIR) region. The choice of those wavelengths is due to the fact that in the NIR region of the spectrum, the absorption of hemoglobin and melanin practically does not affect the variation in skin spectrum while water and lipids become the dominant absorbers and several bands, namely 970, 1200, 1450, 1900 nm correspond to dermal water^36^ (Fig. 3). By design, novel receivers have been made square-shaped with 1.5 mm sides, two silicon detectors replaced with specialized germanium ones, performing adequately in NIR region up to 1800 nm. Center-to-center separation between Light-emitting diode (LED) and Photodetector (PD) has been selected using two configurations of 3.5 mm and 5.5 mm depending on particular pair of LED and PD which takes into consideration light’s mean free path (mpf) in different regions of human skin (Fig. 4b).

In this this work, we utilize GPU-accelerated MC simulation platform^37^. An object-oriented design, along with parallelization through NVIDIA’s CUDA (Compute Unified Device Architecture), enables the model to encode photon-tissue interactions and yield results in real-time. In order to simulate light transport and study sensor signal formation, we utilize a seven-layer optical model of human skin, extensively described in earlier publications^20,21^. Concisely, we consider three major parts: epidermis, dermis and hypodermis which are split into seven functional sub-layers. The optical properties of these layers are described by the specular reflection at the skin-air interface as well as light absorption and scattering therein. We considerably extended the original model into the near infrared range and introduced varying concentrations of chromophores such as melanin $\left({C}_{mel}\right),$ blood $\left({C}_{blood}\right),$ oxygen saturation $\left({S}_{blood}\right),$ as well as the topical water/sweat layer and their corresponding influence on the detected signal at 535, 645, 970 and particularly 1450 nm wavelengths (Fig. 4c).

### Experimental validation of the developed approach

PPG signal produced by SmartWatch sensor largely depends on skin type, its internal properties and external factors such as presence of wrist movement during acquisition of the signal, placement of the device on the wrist, etc. There are also a number of constrains: for instance, the device should not be overtight (i.e. pulling the skin) for the correct operation of sensors. These factors have a lot of flexibility and therefore comprehensive numerical, experimental and data analysis studies in order to determine their margins needs to be performed.

**Figure 5:**
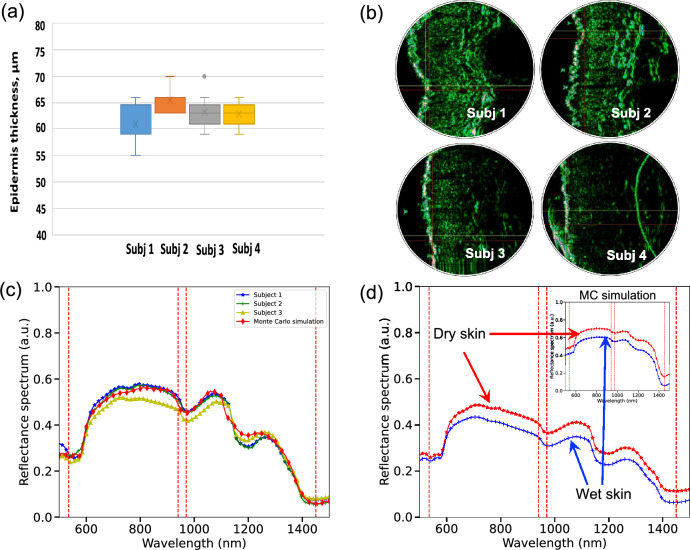
Skin measurements using laboratory grade equipment. An example of evaluated thicknesses (**a**) and ultrasound images (**b**) for test subjects for dorsal surface of the wrists of the left hand; MC simulated human skin reflectance spectra compared with the in vivo measurements for several Caucasian skin types (**c**); MC simulations compared with measurements for wet skin for the entire spectral profile (**d**).

First of all we validated our light transport simulation methods by taking four healthy male and female volunteers and performed several spectral, ultrasonic and SmartWatch skin measurements using laboratory grade equipment. Initially, we captured reflectance spectra of the dry and wet skin of the dorsal surface of the wrists of the left hand. Subsequently, skin thickness has been investigated at the three sites of dorsal surface (medial region) of the wrists of the left hand. Ultrasound images allowed to clearly distinguish the three major functional layers of skin and evaluate their thicknesses, respectively (Fig. 5a, b).

Measuring thickness of skin is an important factor in validation and overall reduction of parameter space. From ultrasonic measurements the mean skin thickness per body site per subject has been estimated. The thickness of the “incoming echo” was considered without taking into account artifacts (hair, air microbubbles) on a smooth skin region with minimal manifestations of deformation, the thickness of which corresponds primarily by the corneal layer of the epidermis. In vivo measurements have been directly utilized in the computer studies of the PPG signal formation of SmartWatch.

**Figure 6:**
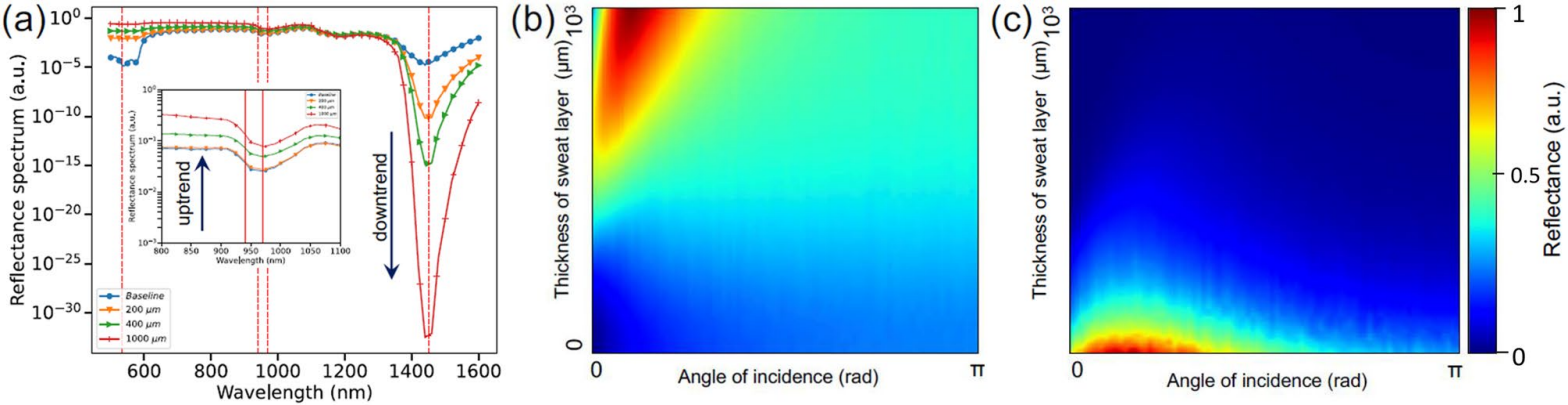
MC simulations. MC simulations and notable trends due to the influence of the increasing thickness of sweat/water film on PPG signal for specific wavelengths of extended SmartWatch sensor (**a**). Complete 2D maps of human skin reflectance for increasing sweat/water layer thickness and the angle of incidence at the wavelengths 970, and 1450 nm (**b**, **c**).

We have mimicked in silico the exact configuration of the developed sensor, the distributions of thicknesses and corresponding optical properties of skin layers. Figure 6a shows several representative cases, where the output of MC simulations and the experimental data has been directly compared for multiple subjects. We achieved excellent match between our computational models and laboratory grade measurements (Fig. 5c, d).

### Identifying features of importance using Monte Carlo and Machine Learning (ML) techniques

Due to excitement, stress, physical exertion, the activity of sympathetic nervous system increases, resulting in the surges of concentration of selected neurotransmitters. The cascades of signal pathways and enzymatic biochemical processes are launched in sweat glands, ensuring the secretion of the sweat fluid^38^. As a result, the sweat film between SmartWatch and wrist skin is formed, which affects the form of a PPG signal. In order to identify the generalized trends, first we have performed MC simulations of the influence of the sweat film thickness between the skin and the PPG sensor (Fig. 6a). Subsequently, simulation of the wrist movement has been performed by changing the angle of incidence of the probing light and combining the increasing thickness of sweat film. With the incidence angle modulation, we were able to estimate a complete set of 2D maps showing the performance of SmartWatch sensor under a variety of detection conditions (Fig. 6b and c).

Several useful trends have been identified both in modeling and measurements: formation of water/sweat film results in the uptrend for 970 nm and corresponding distinct opposite downtrend for 1450 nm. This can be explained by the changing combined skin/water absorption/reflection properties at specific wavelengths. For example, at 970 nm combined PPG signal for wet skin generally (but not always) results in the uptrend compared to baseline whereas 1450 nm exhibits a confident downtrend as it is situated at the known water absorption peak, correspondingly. Apart from water, signal at 970 nm is found to be more sensitive to skin type, its thickness and the effects associated with blood pulsation. Both PPG signals are affected by wrist motion and resulting measurements artifacts.

Nevertheless, transitioning laboratory insights to the measurements obtained with the device in consumer settings remains challenging. Therefore, we performed an advanced analysis of unique dataset (19 human subjects who participated in 103 indoor running trials of 5 km total distance). More information about subjects’ characteristics and ambient conditions can be found in chapter “Materials and methods” and see (ref.^6^).

Diversity of the initial experimental conditions, subject physiology, etc. makes it challenging to determine the exact moment at which sweat film starts to appear. It is not always perceived whether there is sweat under the SmartWatch or not i.e. perception of the sweat on the face is more pronounced then the feeling of sweat on the hand. Moreover, peculiarities of sweating process for each person, speed of sweat saturation of the surface of the skin result in considerable differences in the amount of the liquid under the watch.

With our primary goal is to be able to detect the moment within a certain confidence interval when the film appears using the changes in PPG signal we utilized a simple standard paper sticker method where a paper sensor with embedded dry ink changes color due to sweat presence. In this study we used this as our reference method for determination of the moment of occurrence of a sweat film under smart watch. The sticker sensor was placed on the hand of the runner and positioned under a smart watch. Notably, the exact moment of sweat film occurrence, determined with the sticker sensor, was captured slightly earlier than the runner's personal feeling of sweating. Since all runners were sweating, we investigate the entire PPG dataset throughout the run, to see what generalized changes occur in the signal over the entire run. Physical activity creates several distinct artifacts, mostly related to the movement. We have been extensively looking into these issues and found that PPG signal can easily be corrupted by the combination and influence of several external factors including wrist movement, physical activities, ambient light, ambient temperature and pressure arising from the contact between PPG sensor and skin.

In particular, the influence of wrist movement become more apparent in the case of extremes: e.g. too loose or too tight strap tension of smartwatch. We have monitored the pressure and associated effect for a variety of strap tensions of our smartwatch. Therefore, monitoring those artifacts is crucial for continuous acquisition and we specifically developed a sensor fusion approach to investigate the artifacts resulting from the physical activity, which were mostly related to the movement.

**Figure 7:**
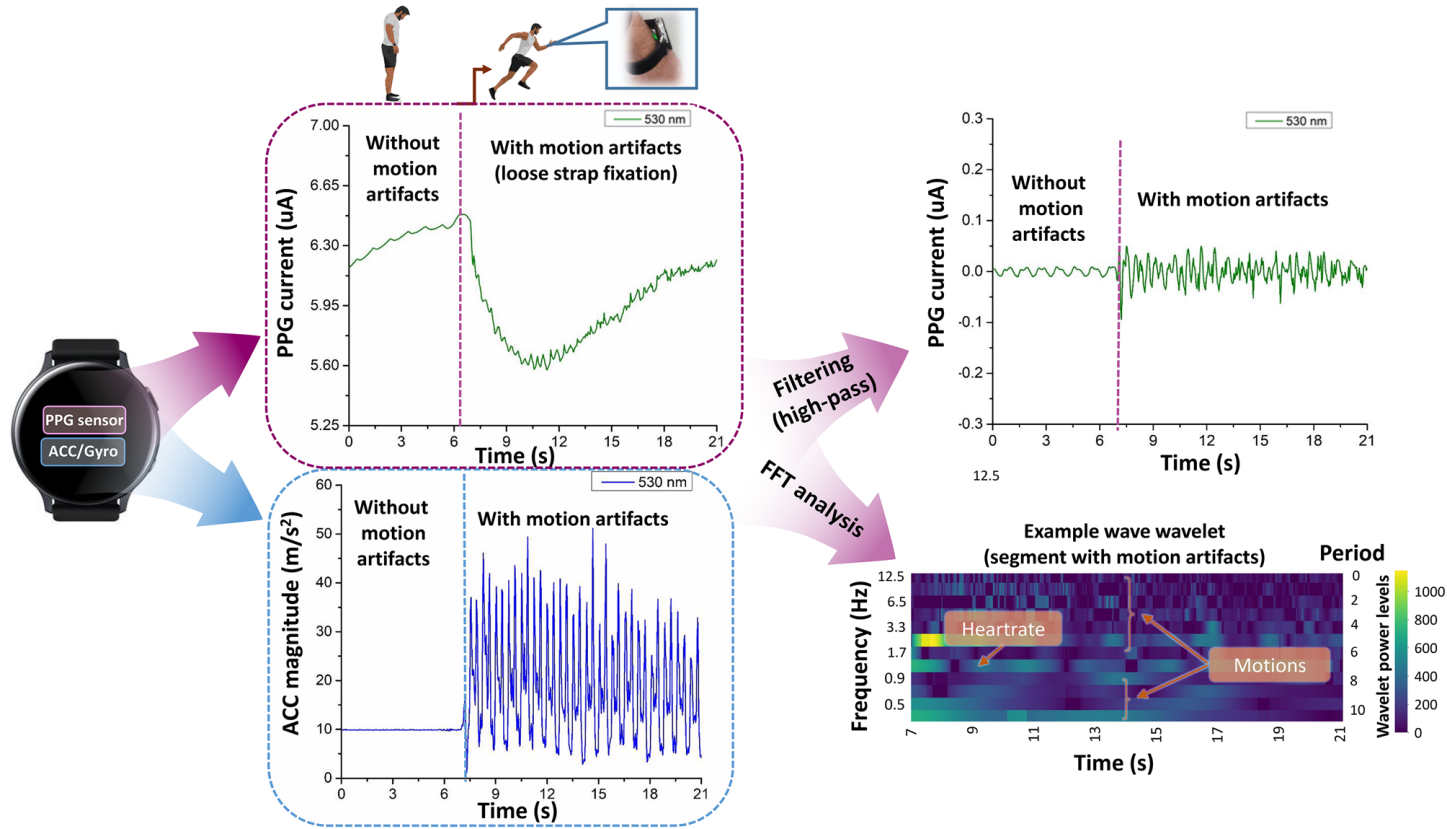
Sensor fusion allows distinguishing various types of artifacts such as motion, movement of arms, steps, heartbeat, etc.

We utilized sensor fusion approach to investigate the artifacts resulting from the physical activity which are mostly related to the movement (Fig. 7). In our SmartWatch the artifacts in the PPG signals are estimated from the IMU (inertial measuring unit) data collected simultaneously with PPG signals. The individual components of PPG signal were investigated by transforming it from time to frequency domain. In order to provide an additional example, we are able to detect distinct frequency corresponding to the heart-beat component in PPG and the others corresponding to a variety of motion artifact contributions.

We noted a high-order correlation with the following factors: running speed, arm swings, tightness of the band, the actual positioning of Smart Watch on the wrist and the body physic of the runner. Likely, the nature of the noise artifacts is not changing, and they can still be quantified by several well-known algorithms. Within a framework of this research, we created a complete capturing, modeling and analysis pipeline (depicted in Fig. 2) specifically tailored to access hydration proprieties of skin with SmartWatch in order to identify the exact moment when sweating occurs. Therefore, we could make several assumptions regarding the key changes occurring during the run that can affect the PPG signal. Firstly, changes in steps per minute (spm) are likely to occur when a person is getting tired, or the test run is commenced. Secondly, the distinct changes in the heart rate beats per minute (bmp) are extremely likely during the runs. Finally, the appearance of sweat is a plausible outcome of an intense exercise. We approach the task by attempting to classify the start and the end of the runs. This task can be represented as a binary classification problem, where the positive class is the end of the run, and the negative class is its start. Subsequently, we test the hypothesis is that if we are able to classify the individual sections of the run with confidence, we can find the signs indirectly indicating the moment of appearance of a sweat film.

**Figure 8:**
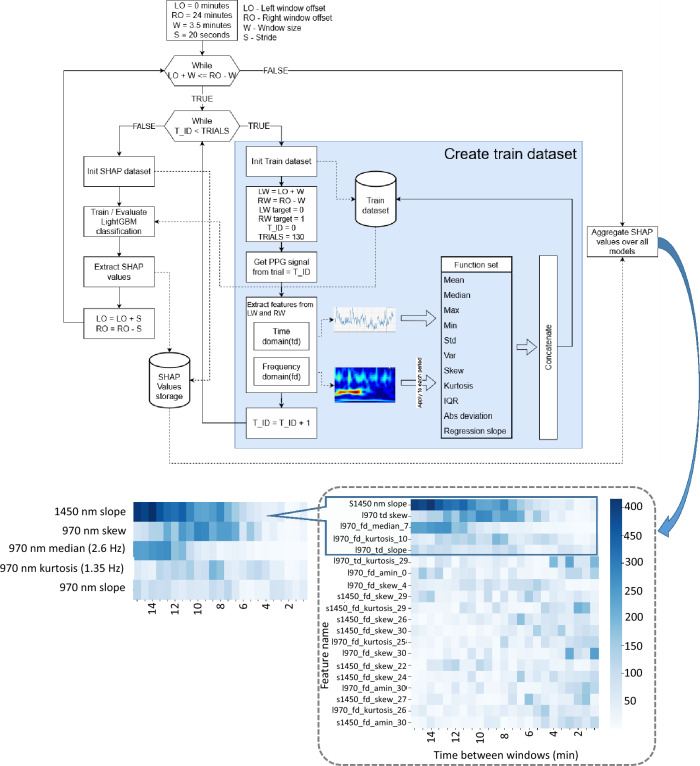
Schematic presentation of workflow used to investigate the quality of ML models for reliable identification of sweat film appearance. A number of prominent models have been obtained with the various quality of classification. Each column on the SHAP chart represents a different LightGBM model, the row represents the top 20 features, and the color represents their importance. Each feature name have name pattern <wave length>_<domain>_<function>_ <channel (for frequency domain only)>.

Following modeling and experimental validation we specifically focused on investigating data features at 970 and 1450 nm wavelengths by training several ML-based classifiers. Our analysis workflow is presented in Fig. 8 and includes several important steps. Firstly, by selecting two windows (3.5 min duration) on the left (start run) and on the right (end run). From each window we extracted time and frequency domain features. Having done this for each user’s run, we assembled a dataset to train the (LightGBM)^39^ classifier where left window represent negative class and right window represent positive class. After we move windows closer to each other by 20 s step, repeated same procedure and train new classifier. We move the windows to each other until they begin to overlap. In total, we have trained 23 classifiers.

**Figure 9:**
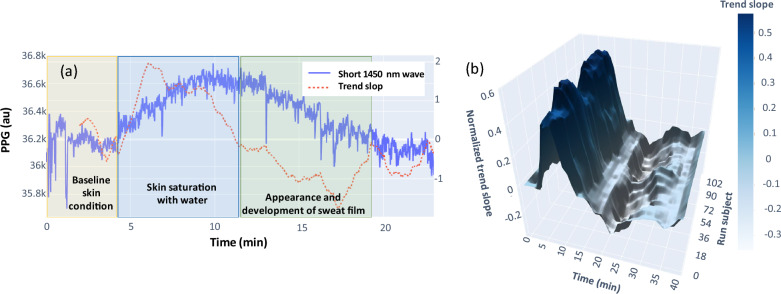
An example of the 1450 nm trend showing the monotonic changes in reflection compared to baseline such as skin saturation, appearance and development of sweat film (**a**). 3D representation of the generalized 1450 nm trend for the entire consumer study (**b**).

On each trained classifier we performed a Shapley Additive Explanations (SHAP)^40^ feature importance analysis. After ranking the features, we selected the 20 most important for analysis (Fig. 9).Time domain features were obtained from completely raw unprocessed signal, frequency domain features involved conversion to the wavelet spectrum. Significance of the following features has been investigated Mean, Median, Max, Min, Std, Var, Skew, Kurtosis, IQR, Median abs deviation, Trend slope. For the time domain data, these functions were applied to the raw signal. For the frequency domain, the functions were applied to each channel of the wavelet transform spectrum. We got a total of 642 features. For the wavelet transformation we used the ssqueezepy library^41^. As a wavelet function we used the Generalized Morse Wavelet^42^. Each LightGBM model has been trained with same parameters max_depth = 2 and learningrate = 0.01 the rest of the parameters were default. To estimate the performance, we used cross validation by 4 folds.

A total of 23 models corresponding to the different time between windows. A high quality of classification has been obtained between windows of more than 15 min resulting in accuracy score values greater than 0.7. This refers to significant differences at the beginning and end of the run. For further study, we selected three main candidate features including: frequency domain features corresponding to the heart rate. For example, the median value of the spectrum on period 7 corresponding to a frequency of 2.6 Hz at a sampling rate of 25 Hz is occupied by the pulse wave. Due to the heart rate changes during test runs, this signal is important in classifying the individual sections of the run. This hypothesis has been extensively evaluated by taking the PPG signal at 970 nm and filtering it from any other frequencies, so we can actually see some analogy to the pulse wave. It confirmed the 970_td_skew feature which has little contribution at the beginning and the end of the run due to the heart rate being stabilized at this regions, similar to the case of 10 min after the start of the run. We assume that both of these features are a consequence of heart rate change during a run.

Our main feature for classification however is the monotonic change in the 1450 nm wavelength signal (1450_nn_slope). We associate this phenomenon with the appearance of a sweat film. The monotonic change fits well with the MC numerical simulations and the fact that the film does not appear instantly. This process is not instant and stretched over time, i.e. transitioning from baseline, skin saturation and at some point sweat film appearing on the skin surface. Evaluating PPG signal at 1450 nm captures these monotonic changes in reflection, respectively.

Figure 9 shows the presence of such trends from 4 to 10 min and from 11 to 20 min associated with the formation of the sweat film.

We can see that starting from the fourth minute, the trend begins its monotonous increase with this process taking place until the 10th minute, subsequently, the trend reverses. In order to determine the trend direction a practical time interval has been selected. We found that the optimal smoothing of the signal is in a window of 2 to 5 min. These time windows allow us to detect quite clearly the appearance of trends during the exercise time. We performed this procedure for all the trials and averaged the values over a window of 5 min and over 5 subjects, and also normalized the value of the slope. With the 3-dimensional graph we can see that on average the positive slope appears from ~ 5 min with peak at ~ 9 min, which changes to negative from ~ 14 min to ~ 20 min. This conforms well with our task of optical detection of sweat film formation within 2-min accuracy window and paves the way for future assessment of sweat body loss and development of a practical applications notifying consumer of the need to rehydrate.

## Discussion

In this paper we considered the task of paramount importance set for implementation in the future generation of wearables: non-invasive continuous assessment of body hydration. For the first time, we demonstrated how this task can be apprehended through sensor fusion approach by extending the capabilities of optical detection and modern analysis algorithms. We presented novel sensing methodology and a pipeline for capturing, modeling and analysis of the skin hydration stages. Performed research provides novel insights on the light-tissue interaction, the multitude of features of importance and industry-led advancement in the work on detection of sweat and body water loss. Our approach allows visualizing the distributions of detected optical signal within tissue for target geometrical, spectral and other sensor configuration as well as performing in silico studies of thin sweat film and its influence on the detected PPG signal. We built prototype device and ML-based solutions for estimation of human skin sweating. Detected trends have been found in good agreement with optical modeling and are supported by theoretical investigation/laboratory studies. Although 1450 nm provides the best SNR for water content estimation, we anticipate that active dehydration (sweating) due to sport activity can be potentially detected with presently available sensors. However, sensing water loss due to a simple luck of intake will require further major investigations.

## Materials and methods

### Datasets

**Figure 10:**
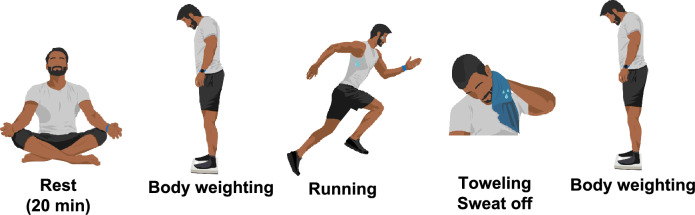
Series of panelist actions during data collection involved rest, weighting before and after test runs while removing sweat with a towel.

For monitoring of human skin hydration and body sweat loss we performed an advanced analysis of user study involving 19 human subjects (male and female, aged 21–52 years) who participated in 103 indoor running trials of 5 km total distance. Specialized test rooms with treadmills and controlled environmental conditions (ambient temperature range 10–34 °C and relative humidity range 25–60%) were prepared for indoor running trials. More information about subjects’ characteristics and ambient conditions can be found in (ref.^5^). All subjects were assumed to have the capacity to fully complete running distances under the specific environmental conditions (preliminary consented). Admission to the trials and control of subject’s individual condition during running trials was supervised by a qualified medical doctor. The data was collected for subjects of Eastern European descent at the Institute of Biomedical Problems, Moscow, Russian Federation. The data collection protocol was reviewed and approved by the Biomedical Ethics Committee of the RF SRC—Institute of Biomedical Problems of the Russian Academy of Sciences/Physiology section of the Russian Bioethics Committee Russian Federation National Commission for UNESCO (Protocol No 541 of May 11, 2020). Committee stated that study protocol aligned with the Declaration of Helsinki. Before the trials, all subjects received detailed explanation of the clinical tests and signed informed consents. The data was kept anonymized, and it was used only for the intended research purpose. According to the protocol each subject has been examined by medical staff and a number of anthropometric parameters (including height) and general information such as age, gender, medical history, exercise habits and current medication has been recorded. After the initial screening, subjects were asked to put on the modified SmartWatch prototype device on the left wrist and perform the following actions (Fig. 10): taking rest (seating) for 20 min in a room with normal temperature (about 23 °C); perform the 1st nude body weighing with precise CAS‑HB‑150 (South Korea) scales; running a 5 km distance with predefined conditions. During the trials the following indicators were continuously recorded: accelerometer, Gyroscope and PPG signals for 535, 940, 970, 1450 nm wavelengths sampled at 25 Hz.

### Hardware prototype of a novel multi-wavelength optical PPG sensor

PPG signals were recorded by developed hardware prototype of a novel multi-wavelength (535, 645, 970 and 1450 nm) optical PPG sensor which had been built into the common Samsung Galaxy Watch Active 2. Receivers had been made square-shaped with 1.5 mm sides, two silicon detectors replaced with germanium ones. LED–PD separation distance had been chosen for two configurations of 3.5–5.5 mm depending on particular pair of LED and PD (Fig. 4b).

### Monte Carlo techniques

Evaluation of the accuracy and the characteristics of PPG sensors has been performed using biomedical optics-based simulation framework with Monte Carlo simulations. We utilized GPU-accelerated MC simulation platform. An object-oriented design, along with parallelization through NVIDIA's CUDA (Compute Unified Device Architecture), enables the model to encode photon-tissue interactions and yield results in real-time. We used workstation with two GeForce RTX 2080 Ti GPU’s (NVIDIA, USA) installed. Each GPU provides 12 GB of onboard RAM and features 4352 CUDA cores. In order to simulate light transport and study sensor signal formation, we utilize a seven-layer optical model of human skin with topical sweat layer.

### Machine Learning techniques

Significance of the features has been investigated Mean, Median, Max, Min, Std, Var, Skew, Kurtosis, IQR, Median abs deviation, Trend slope. Shapley Additive Explanations (SHAP) has been calculated for 30 most important features and their importance (Fig. 8). For the wavelet transformation we used the ssqueezepy library. As a wavelet function we used the Generalized Morse Wavelet. We trained a binary classification model (LightGBM) with the two windows: beginning of the run and the end of the run. LightGBM has been trained with max_depth = 2 and learningrate = 0.01 using cross validation by 4 folds.

### Spectral measurements

The reflectance spectra of four healthy male and female volunteers skin were recorded in the 450–1750 nm range with 1 nm resolution with Cary 5000 Varian spectrophotometer (a high-performance UV–Vis and NIR model). Double beam and a 0°/d measurement geometry has been utilized in our measurements with internal DRA (Diffuse Reflectance Accessory) that includes an integrating sphere of 110 mm diameter. The volunteers did not weare any cosmetic product in the study area (dorsal side of wrist) for at least 24 h and washed their hands 2 h before the measurements were taken. Informed consent has been obtained from all subjects prior to participation in the study with collected data appropriately encoded and subsequently processed. Initially, we captured reflectance spectra of the dry and wet skin of the dorsal surface of the wrists of the left hand. The reflectance spectra were repeatedly acquired at the medial region of dorsal surface of left hand for 3 points in each site located at 2 cm from each other.

### Ultrasonic measurements

The skin thickness had been investigated at the three sites of dorsal surface (medial region) of the wrists of the left hand (3 points in each site located at a distance of 5–7 mm from each other). High-frequency ultrasound DUB SKINSCANNER (tpm taberna pro medicum, Germany) was used with the high-frequency ultrasonic linear water sensor 75 MHz (resolution of 21 microns at the central frequency) as imaging technique. Typical scanogram section method has been utilized to obtain the exact distance values. For instance, dermal thicknesses were obtained by drawing straight lines perpendicular from the skin surface to the dermal—hypodermal junction using Dub Skinscanner Ver 5.1 software. From these measurements the mean skin thickness per body site per subject has been estimated. The thickness of the “incoming echo” was considered without taking into account artifacts (hair, air microbubbles) on a smooth skin region with minimal manifestations of deformation, the thickness of which corresponds primarily by the corneal layer of the epidermis.

## Data Availability

The datasets generated during the current study are not publicly available due to the company rules, but are available from the corresponding author on reasonable request with the permission of Samsung Electronics.
